# Greenhouse Gas Emission Inefficiency Spillover Effects in European Countries

**DOI:** 10.3390/ijerph18094479

**Published:** 2021-04-23

**Authors:** Levent Kutlu, Ran Wang

**Affiliations:** 1 Department of Economics and Finance, University of Texas Rio Grande Valley, Edinburg, TX 77539, USA; 2 Trust Financial Corporation, 303 Peachtree St, Atlanta, GA 30308, USA; wangran2010@gmail.com

**Keywords:** eco-efficiency, greenhouse gas, Kyoto protocol, stochastic frontier analysis, pollution, spillover

## Abstract

In our study, we examine whether spatial spillover effects exist for greenhouse gas emission efficiency for 38 European countries between 2005 and 2014. We find that inefficiencies of other countries would lead to lower efficiency levels for a country. This negative inefficiency spillover effect goes down till 2008 then goes up till 2011, then stays relatively stable after 2011. Any strategy to reduce inefficiencies of other countries could potentially improve the efficiency levels. We find that human development index shows significant positive impact on greenhouse gas emission efficiency levels. In particular, one standard deviation increase in human development index would lead to a 11.12 percentage points increase in the greenhouse gas emission efficiencies on average. Different countries show different efficiency levels and efficiency growth patterns over time. However, the pattern of spatial spillover is quite similar among all countries over time.

## 1. Introduction

Globalization has led to an increase in interactions between countries. As a consequence, the dynamics of the world economy as well as air/water pollution patterns have changed. In particular, the increased linkages between countries placed the governments in a more competitive global market. Generally, the competition is considered to be a good thing, as under some conditions it would increase the social welfare. Moreover, in line with Hicks’ [1] quiet life hypothesis, one may argue that increase in competition would force the firms to work harder in order to achieve higher technical efficiency levels. Hence, given the same level of inputs, the firms can produce more output. The same idea may potentially apply to country level production as well. However, this line of logic ignores the negative externalities in the production process, such as the environmental damage caused by air or water pollution. If some of the countries that fall behind the competition use technologies that are not “environmentally friendly” to catch up with the other countries in terms of their output levels, then competition may also have some negative consequences for these countries. Although globalization can help diffusion of environmentally friendly technologies between countries, replacing the existing technology and/or operating the state-of-the-art technology that is environmentally friendly may be relatively more costly. Hence, the firms and governments would likely show some resistance to adapting environmentally friendly technologies. Environmental inefficiency may also happen if the energy is not used efficiently, as production of energy is one of the important contributors to greenhouse gas emissions (GHG). For example, when we use heating and cooling more than necessary, this would cause environmental inefficiency.

The extensive emission of greenhouse gas is one of the leading factors for global warming problem. The potential future effects of global climate change include more frequent wildfires, longer periods of drought in some regions and an increase in the number, duration and intensity of tropical storms, shrunk glaciers, loss of sea ice, accelerated sea level rise, more intense heat waves, mold infestation, etc. What is more, global health has been impacted negatively by GHG emission. As shown by Haines et al. [2], climate change is an increasing and evolving threat to global health, particularly in low-income countries. GHG emission is widely acknowledged to correlate with child mortality from acute respiratory infections, ischaemic heart disease in adults, and other non-communicable diseases. Hence, it is important to measure and monitor GHG emission efficiency and monitor the spillover effects. The improvement of GHG emission efficiency would help mitigate both environmental and health impacts of GHG emissions, which would help the world to achieve sustainable growth.

International efforts to prevent global warming and reduce greenhouse gas emissions started in the late 1980s. The Kyoto Protocol was established at the third conference of the parties (COP3) on 11th December 1997 to extend the 1992 United Nations Framework Convention on Climate Change (UNFCCC) that commits state parties to reduce greenhouse gas emissions. There are currently 192 parties to the Protocol. The Kyoto Protocol aims to reduce the onset of global warming by reducing greenhouse gas concentrations in the atmosphere to an emission level that would not cause dangerous anthropogenic interference with the global environment system (see Article 2 of the UNFCCC). The impact of Kyoto Protocol on greenhouse gas emission has been extensively tested in many studies. Iwata and Okada [3] find that the Kyoto Protocol has a negative impact on CO_2_ and CH_4_ emissions, no significant impact on N_2_O emissions, and a positive impact on other greenhouse gas emissions. Besides the Kyoto Protocol, the European Union Emissions Trading System (EU ETS) is another factor that affects the emission behavior of European countries, which started in 2005. In 2008, EU ETS regulated the installations, which were responsible for 40% of the European Union’s total greenhouse gas emissions. The scheme has been divided into multiple trading periods. The first three periods are 2005–2007, 2008–2012, and 2013–2020.

In this study, we concentrate on greenhouse gas emission efficiencies of European countries and their spillover effects. In particular, the bad output (greenhouse gas emission) is measured by the total greenhouse gas emissions including land use change and forestry, which is expressed as millions of tons of CO_2_ equivalents. A country is greenhouse gas emission efficient if it produces the minimal amount of greenhouse gas emissions relative to real GDP, i.e., the ratio of GHG emissions and real GDP is minimized. This analysis helps us to understand the different efficiency levels and different efficiency growth patterns over time for different European countries. The spillover effect is the other focus besides GHG emission efficiency in our study. If the countries use “non-environmentally friendly” technologies to achieve better output level during the global competition process, negative spillover will occur as a consequence of such competition. In our analysis, we try to identify whether the spillover effect is positive or negative.

The data envelopment analysis (DEA) and stochastic frontier analysis (SFA) are two commonly used benchmarking approaches in the efficiency measurement literature. The main difference lies in the technique used in these two approaches. To be more specific, the DEA approach relies heavily on mathematical programming methodologies, while the SFA approach employs econometrics techniques to obtain efficiency estimates. Among others, Haynes et al. [4] measure technical efficiency in pollution prevention activities. Emrouznejad et al. [5] examine the optimality of CO_2_ emissions quota in the Chinese manufacturing firms, and Molinos-Senante et al. [6] estimate the potential of reducing GHG emissions for Spanish wastewater treatment plants. Mukherjee [7] finds that the states in India may increase output, while reducing inputs by improving technical efficiencies. Picazo-Tadeo and Prior [8] apply directional distance functions and data envelopment analysis techniques to Spanish ceramic tile producers. Sueyoshi and Wang [9] compare efficiencies between integrated firms and independent firms using DEA environmental assessment. Vlontzos et al. [10] determine efficiency with and without GHG emissions with DEA. Wegener and Amin [11] examine GHG emission efficiency in the oil and gas sector, and Chen and Jia [12] introduce a slacks-based measure model considering undesirable outputs to measure the environmental efficiency of different regions applying DEA methods.

Among others, country-specific GHG emission efficiencies are studied by Herrala and Goel [13], Jin and Kim [14], Robaina-Alves et al. [15], Valadkhani et al. [16], and Kutlu [17]. Except [16], all these studies use variations of stochastic frontier approaches. All of these studies ignore spatial interactions between producers, which may lead to flawed inefficiency estimates. In contrast to these studies, we estimate GHG emission efficiencies of European countries using a spatial autoregressive stochastic frontier model, which captures spatial interactions of producers. Different types of spillover effects in environmental behavior have been summarized in Nilsson et al. [18]. Both positive and negative behavior spillovers have been observed in different studies. Poortinga et al. [19] observe positive spillover, while Klöckner et al. [20] show negative spillover effects from their studies. Additionally, thus, the studies that neglect spillover effects may get biased estimates for efficiency.

We estimate the greenhouse gas emission efficiencies of 38 European countries between 2005 and 2014. Our GHG emission efficiency definition coincides with that of Kutlu [17]. That is, we assume that the countries try to minimize the ratio of GHG emission to real GDP ratio. Even if the objectives of countries are not aligned with this objective, the idea is that we want to measure the extent to which the countries mimic such behavior. In our analysis, we treat human development index (HDI) as a potential determinant for the ratio of GHG emission to real GDP ratio. The HDI was created to emphasize that people and their capabilities should be the ultimate criteria for assessing the development of a country, not economic growth alone. The HDI is the geometric mean of normalized indices for each of the three dimensions: a long and healthy life, being knowledgeable, and having a decent standard of living. As illustrated in Gürlük [21], HDI (which was modified in his study) impacted industrial pollution differently for different countries. Modern (endogenous) growth theory accepts human development, technological progress, and natural resources as the forces behind economic growth. It covers health, education, and economic growth based on its definition and calculation. A higher development level may contribute to the use of environmentally friendly technologies to reduce the industrial pollution and leads to there being more environmentally sensitive individuals in the population. Kyoto Protocol and EU ETS have a positive impact on average efficiency level during the first stage of EU ETS period, 2005–2007, and the beginning of the second period of EU ETS till 2008. However, the efficiency level stays relatively stable during the second period of EU ETS. During the final two years in the second period and the third period of EU ETS, the average efficiency declines from the year 2011. The average efficiency spillover goes down after the launch of Kyoto Protocol and EU ETS during the first period and beginning of second period till the year 2009. However, the efficiency spillover increases throughout the rest of the time periods.

The main difference of our study from the other studies, as we mentioned above, is that our model captures the spatial interactions of countries in terms of their GHG emission efficiencies. Hence, in this study, we are interested in how the European countries interact with each other in terms of their GHG emission efficiency levels. We estimated that the average GHG emission efficiency is 88.7%. Moreover, it turns out that there is a negative inefficiency spatial spillover impact among the 38 European countries during the time period from 2005 to 2014. More precisely, the inefficiencies of other countries lead to additional inefficiency in a country. The negative inefficiency spillover impact prevents some countries from achieving full GHG emission efficiency. By increasing the efficiencies of other countries, it is possible to reach full efficiency levels. The negative spillover effect (in absolute value) goes down from 2005 to 2008 then goes up till 2011, then stays relatively stable after 2011. The CO_2_ trading scheme, which was introduced in 2004, seems to have a positive impact during the initial few years, then the impact decays. HDI turns out to be an important factor that impacts GHG emission efficiencies. In our analysis, one standard deviation improvement for the HDI would lead to a 11.12 percentage points improvement in the average total efficiency. What is more, different countries have a heterogenous efficiency growth pattern over time. However, all countries have the similar spatial spillover pattern over time, regardless of their efficiency levels.

Finally, we briefly summarize earlier studies and their findings to compare our results with the earlier findings. Moreover, ref. [13] finds that relative to 1997, the average CO_2_ emission efficiencies of 177 countries increased in 2007. However, depending on the models they used, the averages of efficiency estimates range between 40% and 64%, which are substantially lower than our estimates. Unlike our model, their models do not consider heterogeneity and spillovers, which may be the reason for the discrepancy between our and their efficiency estimates. Using a variety of different non-spatial econometric methods, ref. [15] estimates GHG emission efficiencies of 26 countries between 2000 and 2011. While we assume a translog functional form, which is flexible, they assume a Cobb–Douglas functional form, which is rarely used in the stochastic frontier literature as it is not a flexible functional form. They find that, compared to 2000–2004 time period between 2005–2011, the efficiencies of Hungary, Slovenia, Portugal, and Ireland improved significantly. Moreover, Sweden, Latvia, UK, Portugal, and Cyprus are the most efficient countries based on their estimates. Using a non-spatial true fixed effects stochastic frontier model, ref. [14] estimates the carbon emission efficiencies of 21 emerging countries between 1995 and 2016. Their efficiency measure concentrates on carbon emission, which differs from ours. Unlike us, they use the Cobb–Douglas functional form for estimations. Their estimates for carbon emission efficiency range between 70.9% and 91.8%. Using a non-spatial stochastic frontier analysis, ref. [17] examined greenhouse gas emission efficiencies of world countries between 1990–2015. In line with our findings, ref. [17] finds that the Kyoto Protocol helped to increase emission efficiency.

The remainder of the paper has been illustrated as follows. Section 2 presents the data and methodology used for this analysis. Section 3 details the results found in this paper. Section 4 provides the conclusion remarks and some policy recommendations.

## 2. Materials and Methods

In order to estimate the GHG emission efficiency, we use a variation of the spatial autoregressive stochastic frontier (SARSF) model suggested by Glass et al. [22]. This model assumes that the inefficiency is an unobserved random variable, which represents the radial distance from the frontier. The model controls for both country-specific heterogeneity and spatial interactions between countries. Below, we provide the details of dataset, estimation methodology, and estimation results.

### 2.1. Data

The production and greenhouse gas dataset is obtained from Kutlu [17]. The GDP and capital (K) input data are collected from the International Monetary Fund (IMF) website (https://www.imf.org/ (accessed on 30 May 2020)). The capital variable is defined as the sum of government, private, and public–private capital stocks. The GDP and capital variables are measured in billions of constant 2011 international dollars. The labor (L) and energy (E) inputs and population (POP) data are collected from the World bank website (https://data.worldbank.org (accessed on 30 May 2020)). The labor input is the total labor force and the energy input is the energy use in billion tons of oil equivalent. The total greenhouse gas emissions (which includes land use change and forestry measured in million tons of CO_2_ equivalents) data are collected from www.climatewatchdata.org (accessed on 30 May 2020).

The distance data is calculated using geographic shortest distance between capital cities based on their latitude and longitude. For each country, the capitol city is used as the base point to obtain latitude and longitude information to simplify the calculation. The data for capital cities’ longitude and latitude come from techslide.com (http://techslides.com/list-of-countries-and-capitals (accessed on 15 February 2021)). The geodist function in SAS software automatically calculates the distance between a pair of cities based on each city’s longitude and latitude. The HDI data are collected from the WHO (World Health Organization, Geneva, Switzerland) website (https://gateway.euro.who.int/en/indicators/hfa_42–0500-undp-human-development-index-hdi/ (accessed on 8 March 2021)). A detailed calculation of HDI is offered by UNDP (United Nations Development Programme) (http://hdr.undp.org (accessed on 8 March 2021)). HDI is a comprehensive measurement of the average achievements in a country in three basic dimensions of human development: a long and healthy life, access to knowledge, and a decent standard of living. It relates to health, education, and economic growth based on its definition and calculation. We consider HDI as a potential determinant of efficiency.

The final dataset includes 38 European countries for the years between 2005 and 2014. We started with a dataset that has 44 countries. Three countries (Latvia, Montenegro and Serbia) are dropped from the dataset due to missing data or negative GHG values during the 2005 to 2014 time period. Three more countries (Moldova, North Macedonia, and the United Kingdom) are further dropped because of missing HDI data. Total number of observations used in the analysis is 380, 38 countries each with 10 years’ annual data. We present the descriptive statistics of our dataset in Table 1. As shown in Table 1, GHG shows wide variations (i.e., standard deviations are larger than the means); the average GHG is 188.813, while the 5th percentile is as low as 3.905 and 95% goes up to 808.115. We also observe large variations from GDP, POP (Population), L (Labor Input), E (Energy Input), and K (Capital Input). HDI and DIST (Distance) show relatively smaller variations compared to the other variables (i.e., standard deviations are smaller than the means).

### 2.2. Econometric Model

Conventional stochastic frontier models do not control for heterogeneity and spatial spillovers. In the panel data context, Greene [23,24], Wang and Ho [25], Kutlu and McCarthy [26], and Kutlu et al. [27,28] criticize the models that do not disentangle heterogeneity and efficiency, as the heterogeneity can be confused with inefficiency. In this study, we control heterogeneity by country-fixed effects.

Glass et al. [29,30] present distribution-free spatial SFA models. However, Kutlu [31,32] argues that distribution-free SFA models may have robustness issues when there are outliers, unless these outliers are carefully handled. In line with the commonly used SFA models, Glass et al. [22] suggest an alternative spatial SFA model, which assumes a distribution for the inefficiency term. In particular, Glass et al. [22] consider a spatial autoregressive stochastic frontier (SARSF) model. An alternative spatial SFA model may directly model the inefficiency term as a spatial autoregressive random variable. We would rather follow the model of [22] (up to minor modifications). We assume the following model:

(1) $$y_{it}=\alpha_{i}+\rho \sum_{j}w_{ij}y_{jt}+x_{it}^{\prime }\beta +v_{it}+u_{it},$$

where $y_{it}=\ln\left(GHG_{it}/GDP_{it}\right)$ is the logarithm of the ratio of GHG emissions and real GDP for country i at time t; $\alpha_{i}$ is the country-specific fixed effects; $x_{it}$ is the vector of frontier variables, which are exogenous, including labor input, capital input, energy input and population; $v_{it}\sim N\left(0,\sigma_{v}^{2}\right)$ is the usual two-sided error term; $u_{it}=\exp\left(z_{it}^{\prime }\gamma \right)u_{it}^{*}$, where $u_{it}^{*}\sim N^{+}\left(0,1\right)$ and $z_{it}$ is the vector of variables that explains GHG emission efficiency, which is exogenous; $w_{ij}\geq 0$ is the exogenous spatial weight for the effect of $j^{th}$ country’s GHG emissions to real GDP ratio (i.e., $y_{it}$) on the GHG emission to real GDP ratio of $i^{th}$ country and $w_{ii}=0$, which rules out self-spillover. Here, $N\left(0,\sigma_{v}^{2}\right)$ and $N^{+}\left(0,1\right)$ denote the normal and half-normal distributions, respectively. We assume that $v_{it}$ and $u_{it}^{*}$ are independently and identically distributed. In this model, the SAR term, $\sum_{j}w_{ij}y_{jt}$, captures the total spillovers on the $i^{th}$ country from the other countries. More precisely, $w_{ij}$ is the weight that represents the relative spillover effect of $i^{th}$ country on $j^{th}$ country. Following Glass et al. [22], we assume that the exponential weighting matrix W (with elements $w_{ij}$) is row-normalized so that $\sum_{j}w_{ij}=1$. Kutlu [33] proves that if ρ∈[0,1) and W is a row-normalized matrix, inefficiency, $u_{it}$, cannot be negative. This would assure that efficiency lies in the unit interval. Kutlu et al. [28] prove a similar theorem for scalar-normalized weighting matrices. In particular, they show that if ρ∈[0,1) and the normalizing constant lies in a specific interval, the inefficiency would be non-negative. In matrix notation, our model would be:

(2) $$y_{.t}=\rho Wy_{.t}+X_{.t}\beta +u_{.t}+v_{.t}$$

where $y_{.t}={\left(y_{1t},y_{2t},\ldots ,y_{Nt}\right)}^{\prime }$, $u_{.t}={\left(u_{1t},u_{2t},\ldots ,u_{Nt}\right)}^{\prime }$, $v_{.t}={\left(v_{1t},v_{2t},\ldots ,v_{Nt}\right)}^{\prime }$, and $X_{.t}={\left(x_{1t},x_{2t},\ldots ,x_{Nt}\right)}^{\prime }$. The parameters of this model cannot be estimated using the conventional stochastic frontier methods due to endogeneity of the SAR term, $\rho Wy_{.t}$. Consistent parameter estimates can be obtained by estimating the following transformed model:

(3) $$y_{.t}={\tilde{X}}_{.t}\beta +{\tilde{u}}_{.t}+{\tilde{v}}_{.t},$$

where ${\tilde{X}}_{.t}={\left(I_{N}-\rho W\right)}^{-1}X_{.t}$, ${\tilde{u}}_{.t}={\left(I_{N}-\rho W\right)}^{-1}u_{.t}$, and ${\tilde{v}}_{.t}={\left(I_{N}-\rho W\right)}^{-1}v_{.t}$.

We estimate the inefficiency term by:

(4) $${\hat{u}}_{it}=E[u_{it}|\varepsilon_{it}],$$

where $\varepsilon_{it}=v_{it}+u_{it}$ is the composed error term. In practice, we replace $\varepsilon_{it}$ by ${\hat{\varepsilon }}_{it}=y_{it}-{\hat{\alpha }}_{i}-\hat{\rho }\sum_{j}w_{ij}y_{jt}-x_{it}^{\prime }\hat{\beta }$, where ${\hat{\alpha }}_{i}$, $\hat{\rho }$, and $\hat{\beta }$ are the corresponding parameter estimates.

Unlike the conventional stochastic frontier models, β parameters are not the marginal effects. The total marginal effect of $k^{th}$ frontier variable is defined as the marginal change in $y_{it}$ as a response to changes in $x_{kjt}$ for all j:

(5) $$\sum_{j}\frac{\partial y_{it}}{\partial x_{kjt}}=\beta_{k}\sum_{j}{\left[{\left(I_{N}-\rho W\right)}^{-1}\right]}_{ij},$$

where ${\left[.\right]}_{ij}$ represents $ij^{th}$ component of a matrix. The efficiency is estimated by:

(6) $$EFF_{it}=exp(-{\tilde{u}}_{it}),$$

where ${\tilde{u}}_{.t}={\left(I_{N}-\hat{\rho }W\right)}^{-1}{\hat{u}}_{.t}$.

Kutlu [33] defines the direct inefficiency of $i^{th}$ country as the part of the inefficiency that results due to reasons other than spillovers. Similarly, the indirect inefficiency is defined as the part of the inefficiency that is resulting only from spillovers of other countries. Direct and indirect inefficiencies are given by:

(7) $$\begin{array}{c} IE_{it}^{dir}={\left[{\left(I_{N}-\rho W\right)}^{-1}\right]}_{ii}u_{it}\\ IE_{it}^{ind}=\sum_{i\neq j}{\left[{\left(I_{N}-\rho W\right)}^{-1}\right]}_{ij}u_{jt}. \end{array}$$

The counterfactual efficiency difference between no spatial spillovers and spatial spillovers scenarios is given by:

(8) $$\Delta Eff=\exp\left(-IE_{it}^{dir}\right)-\exp\left(-\left(IE_{it}^{dir}+IE_{it}^{ind}\right)\right).$$

**Figure 1:**
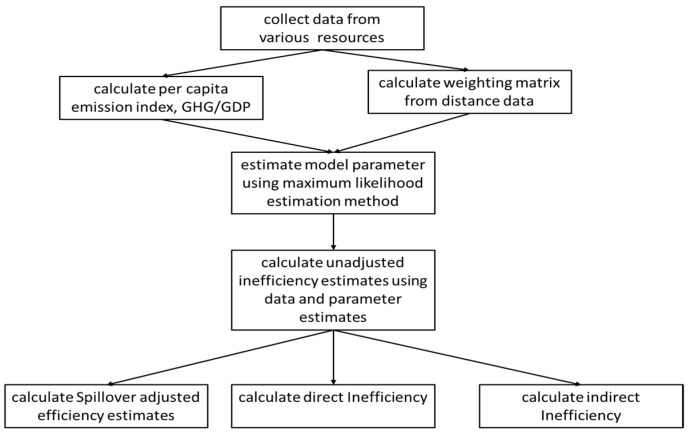
Model Flow Chart.

The methodology scheme is demonstrated in Figure 1. The flow chart shows the whole sequence of phases, tools and results at each stage. After collecting the data from various data sources, we calculated per capita emission index by GHG/GDP and calculated the exponential weighting matrix from distance data. The model is estimated via the maximum likelihood estimation method using the SARSF model. The unadjusted inefficiency estimates are calculated from the data and parameter estimates. Using the above equations, we further calculated the spillover adjusted efficiency estimates, direct inefficiency, and indirect inefficiency.

### 2.3. Empirical Model

We assume that the GHG to real GDP ratio is a function of three inputs: labor, capital, and energy. More precisely, we model the GHG to real GDP ratio via a translog functional form, which may be considered as a second-degree Taylor series approximation to an unknown functional form. We also include the logarithm of population as a control variable. The population indicates the size of the country. Country-specific heterogeneity is controlled via country fixed effects for both the emission efficiency frontier and inefficiency. Similarly, we controlled heterogeneity in efficiency via country-specific dummy variables. We also model the distribution of the GHG emission inefficiency as a function of HDI. This would enable us to examine whether more developed countries are more efficient or not. Time trend variables are also controlled in the empirical model to allow for time trends.

## 3. Results

We present the estimation results for GHG emission efficiency using our spatial autoregressive stochastic frontier model in Table 2. All parameter estimates and heterogeneity tests for both frontier and inefficiency terms are statistically significant at any conventional significance levels. Statistical significance of the SAR term suggests that spatial spillovers exist for GHG emission efficiency. The mean and median GHG emission efficiency estimates for the whole sample are 88.67 and 93.98, respectively. Hence, although the average efficiency of the 38 European countries is reasonably high, there is still room for improvement. Indeed, we predict that the mean and median of total efficiency improvements in response to 1 standard deviation improvement for the HDI are 11.12 and 6.02 percentage points, respectively. That is, if all the countries in the sample increase their HDI by one standard deviation, the average and median of GHG emission efficiencies would increase by 11.12 and 6.02 percentage points, respectively. More developed countries tend to have higher GHG efficiency levels. On the other hand, the mean and median direct efficiency increases in respond to 1 standard deviation increase in HDI for the relevant country are 10.07 and 5.89, respectively. Hence, the development levels of nearby countries affect the GHG emission efficiency levels, but at a limited capacity.

In Table 3, we present the country-specific averages of efficiency estimates over the time period 2005 to 2014. Spain has the highest average efficiency level of 97.02, followed by Italy, France, Hungary, Austria, Malta, the Czech Republic, Germany, and Belgium, which have efficiency levels higher than 96. Sweden has the lowest level of efficiency, around 29. Finland, Estonia, Slovenia, Croatia, and Georgia have relatively low efficiency levels, which are lower than 80. Different countries have different emission efficiency levels. Future environmental policies might focus more on the countries that have lower efficiency levels and their industry composition to make adjustments. By doing this, the overall efficiency level could respond better to environmental policies.

The inefficiencies of the other countries would lead to higher inefficiency in a country, which has a negative impact for the country. The magnitude of spillover effect is heterogenous for different countries. Norway, Russia, and Lithuania are impacted most significantly, which have negative spillover effects of more than 4. Country like Sweden has the minimal spillover effect at 0.87, compared with other countries. Therefore, the low efficiency level for Sweden is not caused by negative spillover.

**Figure 2:**
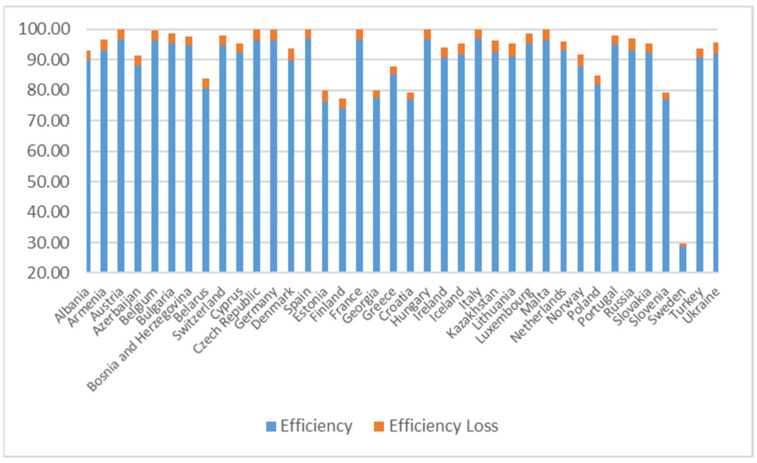
Average Efficiency and Average Efficiency Loss by country.

The average efficiency and average efficiency spillover effect are given by Figure 2. The blue bar shows the average efficiency level for each country and the orange bar shows the average spillover effect for each country. Basically, the orange bar represents the efficiency loss due to other countries being inefficient. Stacking the blue bar and orange bar together, we are able to visualize the potential possible efficiency level without such negative spatial spillover impact. This can be achieved when all other countries achieve full efficiency. From Figure 2, several countries, including Austria, the Czech Republic, Germany, Spain, France, Hungary, Italy, and Malta could reach full efficiency level if there were no negative spatial spillover effects. Strategies that reduce the negative spatial spillover impact would help to improve the efficiency level for these countries. That is, strategies that improve the efficiency levels in other countries or propagation of negative effect of inefficiency from other countries would help to improve the efficiency in the relevant country. Hence, agreements such as Kyoto Protocol and EU ETS would be beneficial through potential spillover effects.

**Figure 3:**
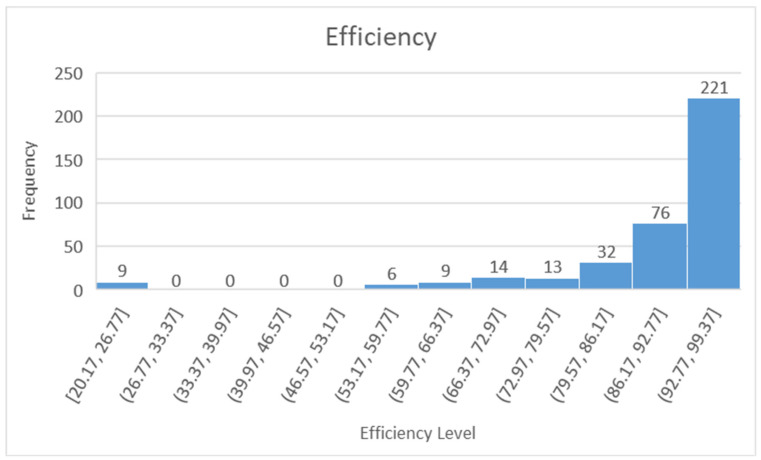

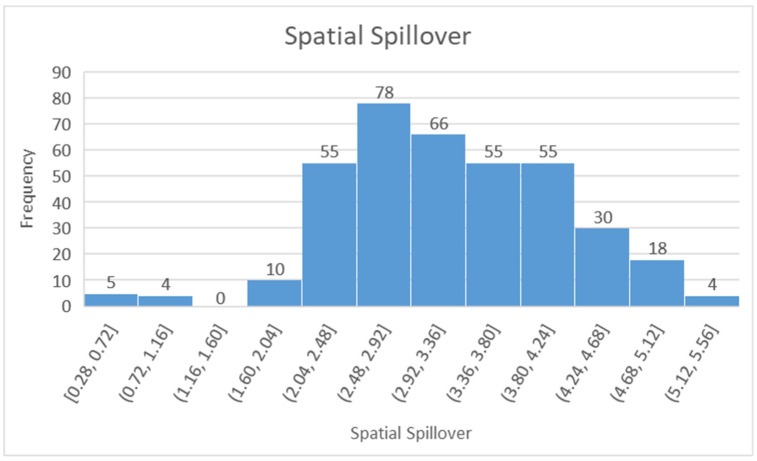
Efficiency and Spatial Spillover distribution.

The distribution of efficiencies and spillovers are given in Figure 3. The majority of efficiencies remain higher than 86%. However, Sweden has very low efficiency levels in some years, which is lower than 30. We believe that Sweden may be an outlier. In Figure 3, on the *x*-axis, we give the range of efficiency and efficiency spillover levels, and on the *y*-axis, we give the frequency of efficiencies and efficiency spillovers that lie in the corresponding interval.

**Figure 4:**
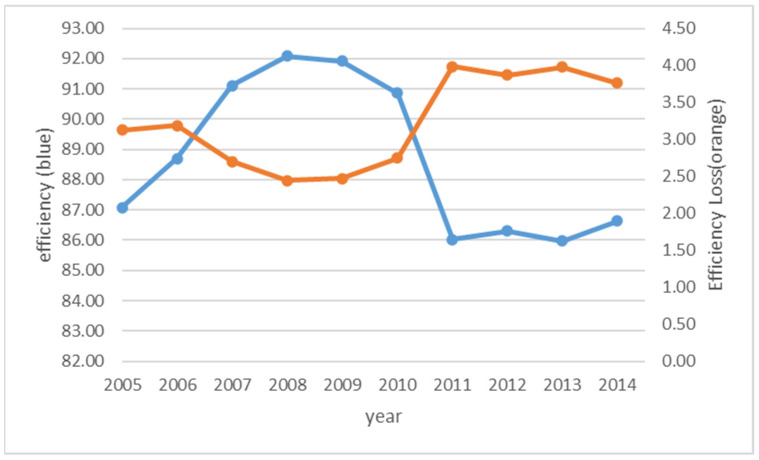
Efficiency and efficiency spillover by year.

The average efficiency and average efficiency spillover by year are given in Figure 4. As shown in Figure 4, the average efficiency level goes up in the first few years till 2008, then stays relatively stable, then goes down quickly, then stays stable after 2011. The negative spillover goes down till 2008 then goes up till 2011, then stays relatively stable after 2011. Based on this observation, we see that the average efficiency is not consistently improving over time. At the beginning of 2005, the European Union member countries launched EU ETS. In line with this, the GHG emission efficiencies have improved after initial few years since EU ETS. However, the impact does not last after 2008.

**Figure 5:**
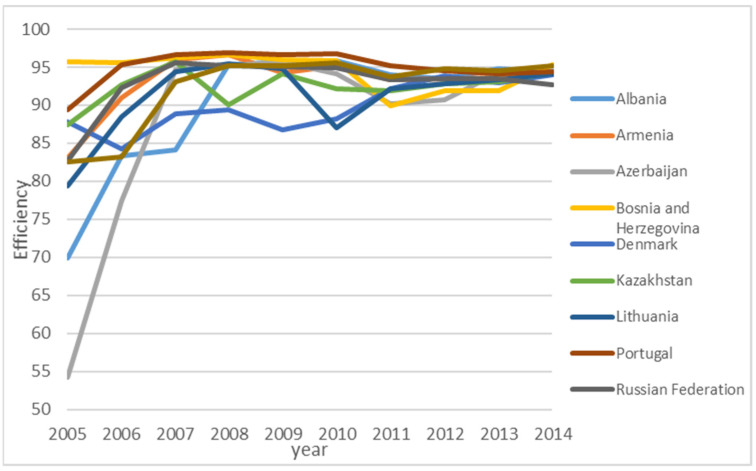
Countries with consistent efficiency improvement over time.

**Figure 6:**
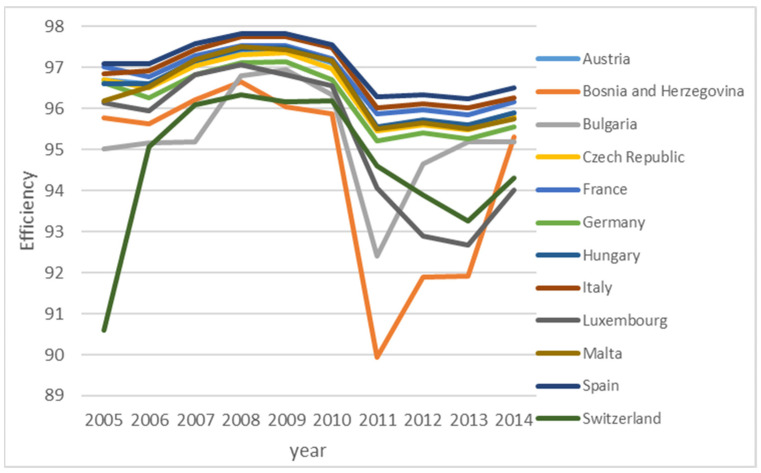
Countries with S shape efficiency.

**Figure 7:**
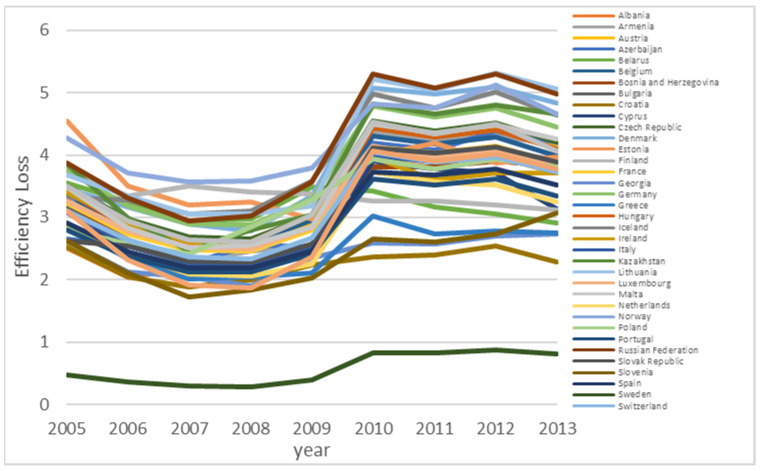
Spatial Spillover for all countries over time.

Detailed analysis shows different efficiency improvement patterns for different countries. Nine countries (Albania, Armenia, Azerbaijan, Denmark, Kazakhstan, Lithuania, and Portugal) show a consistent improvement of efficiency level over time, as shown in Figure 5. For these nine countries, EU ETS helps to improve the efficiency of GHG emission. Twelve countries (in Figure 6) show improvement right after the EU ETS scheme was introduced, but the efficiency level drops down to a certain level, then stays relatively stable afterwards. For these countries, the impact of EU ETS is not persistent. Other countries either have stable efficiency levels or do not have a strong pattern. The finding is that the EU ETS scheme works differently for different countries. However, the EU ETS scheme shows similar spatial spillover patterns for all 38 countries over time, as shown in Figure 7. To be more specific, in the initial few years, we observe a decrease in negative spatial spillover impact, but such negative spatial spillover starts to increase after 2008, then stays relatively stable after 2011. Since there are many countries, the figures are provided to give some idea about the general pattern, rather than presenting individual efficiency levels of specific countries.

## 4. Conclusions

In this paper, we examined the GHG emission efficiency spillover effects of 38 European countries between 2005 and 2014. In our analysis, we find that GHG emission inefficiency of a country would be positively related to inefficiencies of other countries. Hence, other countries cause negative efficiency spillovers through their inefficiency. The countries could have achieved better efficiency levels without such negative spatial spillover effects. To be more specific, some countries such as Austria, the Czech Republic, Germany, Spain, France, Hungary, Italy, and Malta could have reached full efficiency level if there were no negative spatial spillover effects, which is possible when all other countries reach full GHG emission efficiency levels. Hence, agreements related to the environment might be beneficial through not only direct efficiency improvements but also indirect efficiency improvements via spatial spillovers. For example, our findings are consistent with the aims of the Kyoto Protocol and the EU ETS, in the sense that overall, these programs led to GHG emission efficiency improvements for the European countries that we studied. In our study, the dataset that we have did not allow us to disentangle exactly what parts of these programs helped efficiency improvements most. In future studies, it might be worth it to examine the effects of specific characteristics of these programs to reveal most effective strategies that may help to improve the GHG emission efficiencies of countries. Nevertheless, as mentioned above, we deduce that combining forces via agreements not only has a direct positive effect in terms of GHG emission efficiency, but also this has an indirect positive effect through efficiency spillovers by reducing other countries’ inefficiency.

Although the average efficiency of the 38 European countries is reasonably high, there is still room for improvement. HDI is one of the important factors that impacts the inefficiency level. Based on our study, if the HDI increases by 1 standard deviation for all the countries in the sample, the average GHG emission efficiencies would increase by 11.12 percentage points. Government policies that improve HDI could benefit GHG emission efficiency. Since HDI is a combined index for a long and healthy life, being knowledgeable and have a decent standard of living, any policy that improved the medical system, physical well-being, education system and economic level would benefit HDI.

## Figures and Tables

**Table 1:** Descriptive statistics.

Variable	Unit	Mean	Std. Dev.	5th Perc.	Median	95th Perc.
GHG	Million tons	188.813	408.999	3.905	60.240	808.115
GDP	Billion dollars	547.138	845.318	13.607	236.158	2778.353
L	Million	9.301	14.568	0.199	4.246	41.435
K	Billion dollars	1198.112	1879.310	26.304	554.625	5999.062
E	Million kg oil equivalent	4.785	16.393	0.002	0.196	24.862
POP	Million	19.470	29.543	0.430	8.208	80.350
HDI		0.833	0.066	0.717	0.849	0.923
DIST	1000 km	1.131	0.648	0.278	1.028	2.377
Number of Observations		380				

**Table 2:** SAR Stochastic Frontier GHG Emission Efficiency Model Estimates.

ln(GHG/GDP)	Coeff.	S.E.	*p*-Value	
ln(L)	2.85389	0.00060	0.00000	***
ln(K)	0.68344	0.00017	0.00000	***
ln(E)	−1.17232	0.00402	0.00000	***
T	−0.05541	0.00020	0.00000	***
0.5 × ln(L)^2^	−0.54381	0.00045	0.00000	***
0.5 × ln(K)^2^	−0.04605	0.00007	0.00000	***
0.5 × ln(E)^2^	−0.24015	0.00146	0.00000	***
0.5 × T^2^	−0.00172	0.00014	0.00000	**
ln(L) × ln(K)	−0.27515	0.00030	0.00000	***
ln(L) × ln(E)	0.43111	0.00085	0.00000	***
ln(L) × T	0.02596	0.00099	0.00000	***
ln(K) × ln(E)	0.10469	0.00143	0.00000	***
ln(K) × T	−0.00075	0.00004	0.00000	***
ln(E) × T	−0.01208	0.00034	0.00000	***
ln(POP)	−0.23533	0.00096	0.00000	***
County Dummies	YES			
ρ	0.25058	0.00078	0.00000	***
σ_v_				
Constant	−6.95784	0.00071	0.00000	***
σ_u_				
HDI	−95.66927	0.14984	0.00000	***
T	−0.23830	0.08162	0.00350	*
0.5 × T^2^	0.12711	0.02021	0.00000	***
County Dummies	YES			
Average Efficiency	88.67			
Median Efficiency	93.98			
Log-likelihood	584.08			

Note: * *p*-value < 0.01, ** *p*-value < 0.001, and *** *p*-value < 0.0001.

**Table 3:** Average Efficiency Estimates and Effects of Spillovers.

Country	Efficiency	Efficiency Loss	Country	Efficiency	Efficiency Loss
Albania	90.11	2.89	Croatia	77.03	2.33
Armenia	93.10	3.45	Hungary	96.52	3.48
Austria	96.51	3.49	Ireland	90.63	3.22
Azerbaijan	88.10	3.16	Iceland	91.58	3.88
Belgium	96.20	3.41	Italy	96.85	3.15
Bulgaria	95.29	3.22	Kazakhstan	92.42	3.85
Bosnia and Herzegovina	94.52	3.16	Lithuania	91.18	4.00
Belarus	80.69	3.16	Luxembourg	95.29	3.29
Switzerland	94.64	3.15	Malta	96.43	3.56
Cyprus	92.21	3.05	Netherlands	93.14	2.87
Czech Republic	96.42	3.58	Norway	87.67	4.14
Germany	96.20	3.80	Poland	81.73	3.12
Denmark	89.84	3.87	Portugal	95.02	2.84
Spain	97.02	2.98	Russia	92.86	4.09
Estonia	76.32	3.57	Slovakia	92.31	3.12
Finland	74.04	3.39	Slovenia	76.80	2.47
France	96.71	3.29	Sweden	28.72	0.87
Georgia	77.56	2.48	Turkey	90.61	3.08
Greece	85.18	2.59	Ukraine	91.95	3.57

## Data Availability

Datasets that we use are publicly available on stated websites.
